# Flash Flood Early Warning System in Colima, Mexico

**DOI:** 10.3390/s20185231

**Published:** 2020-09-14

**Authors:** José Ibarreche, Raúl Aquino, R. M. Edwards, Víctor Rangel, Ismael Pérez, Miguel Martínez, Esli Castellanos, Elisa Álvarez, Saul Jimenez, Raúl Rentería, Arthur Edwards, Omar Álvarez

**Affiliations:** 1 Faculty of Telematics, University of Colima, 333 University Avenue, Colima C.P. 28045, Mexico; aquinor@ucol.mx (R.A.); ismael_perez@ucol.mx (I.P.); esli_castellanos@ucol.mx (E.C.); maaguilar@ucol.mx (E.Á.); arted@ucol.mx (A.E.); xe1aom@ucol.mx (O.Á.); 2 School of Mechanical, Electrical and Manufacturing Engineering, Loughborough University, Wolfson Building, Ashby Rd, Loughborough LE11 3TU, UK; R.M.Edwards@lboro.ac.uk; 3 UNAM, Ciudad Universitaria, Mexico City D.F. 04510, Mexico; victor@fi-b.unam.mx; 4 Faculty of Civil Engineering, Carretera Colima-Coquimatlán km 8.5, Coquimatlán C.P. 28400, Colima, Mexico; martinez_miguel@ucol.mx; 5 Corporativo STR S.A. de C.V., 111 Canario Street, Colima C.P. 28017, Mexico; saul.jimenez@corporativostr.com; 6 Tairda S.A. de C.V., 111-B Canario Street, Colima C.P. 28017, Mexico; raul.renteria@tairda.mx

**Keywords:** real-time early warning, flash flooding, internet of things

## Abstract

This paper presents a system of sensors used in flash flood prediction that offers critical real-time information used to provide early warnings that can provide the minutes needed for persons to evacuate before imminent events. Flooding is one of the most serious natural disasters humans confront in terms of loss of life and results in long-term effects, which often have severely adverse social consequences. However, flash floods are potentially more dangerous to life because there is often little or no forewarning of the impending disaster. The Emergency Water Information Network (EWIN) offers a solution that integrates an early warning system, notifications, and real-time monitoring of flash flood risks. The platform has been implemented in Colima, Mexico covering the Colima and Villa de Alvarez metropolitan area. This platform consists of eight fixed riverside hydrological monitoring stations, eight meteorological stations, nomadic mobile monitoring stations called “drifters” used in the flow, and a sniffer with data muling capability. The results show that this platform effectively compiles and forwards information to decision-makers, government officials, and the general public, potentially providing valuable minutes for people to evacuate dangerous areas.

## 1. Introduction

Amongst natural disasters, floods represent one of the great global challenges harming humankind. This phenomenon leaves tens of thousands of victims worldwide. In terms of lives lost and property damaged, floods are ranked just behind tornadoes as the top natural disaster in the United States. According to studies by the United Nations Office for Disaster Risk Reduction (UNDRR) [1], since 1995, floods represented 47% of all weather-related disasters and are continuously listed first among natural disasters worldwide. In recent years (2008–2017), approximately 73.1 million people were affected by floods. The total number of people affected grew significantly in 2018 to 34.2 million people [2]. The data show a trend that the number and severity of floods may be increasing, thus making it more important for persons to better anticipate floods, according to experts within the United Nations Climate Change Conference (COP21). As the phenomenon of a dynamic range of energy in weather systems increases, effects are increasingly noticeable in the hydrological cycle and associated river system catchment areas.

Flood prediction models may be tuned using historical hydrological data concerning fluvial floods [3,4]. However, due to climate change and the increased levels of energy in weather systems, flash flooding is becoming more common and therefore, when considering flood alerting systems, historical data are less useful than real-time data [5,6]. Flash floods concentrate their waters in small geographic areas within six hours of the rains or other events that spawned them and are characterized by a rapid rise of fast-moving water. Water moving at 10 m per second, a common speed for flash floods, can move rocks weighing almost a hundred pounds. Flash floods carry debris that elevates their potential to damage structures and injure people.

The rest of this paper is laid out as follows. This section presents a short review of systems of sensors with similar applications to EWIN (Emergency Water Information Network). Section 2 shows materials and methods, Section 3 analyses the results, Section 4 presents a particular discussion, and Section 5 reports our conclusions.

Several related projects are currently under development around the world. The authors in [6] and [7] present the development of the European Flood Warning System (EFAS), which aims at increasing preparedness for floods in trans-national European river basins by providing local water authorities with medium range and probabilistic flood forecast information, 3 to 10 days in advance. In Part 1, the approach used is based on quantiles. The core of EFAS consists of a grid-based distributed hydrological rainfall–runoff model with a routing component that simulates the hydrological processes in large river basins. This model is fed with several medium range weather forecasts, including full sets of the Ensemble Prediction System (EPS). Part 2 concerns the assessment of EFAS overall forecasting over a full two-year period of existing EFAS operational hydrological forecasts.

In [8], the authors apply a regional flood frequency analysis on a global scale, exploring its suitability as a means of providing a preliminary characterization of flood behavior, while also identifying behavioral patterns between different catchments. The results suggest that regional flood frequency methods applied to suitably partitioned catchment groups can provide plausible discharge estimates on a global scale.

The system in [9] uses rainfall intensity data from terrestrial microwave communication links and the geostationary Meteosat Second Generation (MSG) satellite.

Based on publicly available meteorological data, this provides early warning through location-based assessments of precipitation forecasts and optional flood forecasts. The software is made up of the following components:

(a) Mobile Smartphone Application and VGI (Volunteered Geographic Information) data: Volunteers can share photographs, water levels, classification of the precipitation, and estimations of snow depths. The application runs on the Android operating system.
(b) Forecasting System: The applied meteorological data originate from the free open data platform of the German Weather Service (DWD). The operational flood forecast is based on a model available from the DWD; periodically, the systems checks for enough VGI measurements to update the model state.
(c) Web interface (dashboard): Allows authorized crisis managers to conveniently monitor the situation in the catchment area, map visualization, hydrological, and meteorological forecasts, and warnings as well as the user-generated VGI data.
(d) Server: Based on a JavaScript runtime environment with a Node.js web server in combination with the Express.js web framework and a MongoDB NoSQL database. Vue.js is used for the frontend of the web framework and Google Firebase Cloud Messaging service is used to dispatch push notifications to the mobile end devices [10].

FLOODIS is a cloud-based flood emergency system which is composed of different subsystems that work independently; the main components are:

(a) GEO Gateway: Interfaces with all existing European Emergency Systems like Copernicus EMS and EFAS to provide flood extent and flood forecast maps.
(b) Multi-platform Mobile Application: Sends real-time geolocated flood user reports, receives terrain maps together with additional layers and nearby user reports, and receives flood-related alerts from the authorities via FLOODIS.
(c) Augmentation service: Receives GNSS (Global Navigation Satellite System) data from the mobile application and computes an augmented and validated GNSS position, featuring increased accuracy and integrity.
(d) Service layer: Acts as the FLOODIS centralized service provider, implementing web services to receive/provide geolocated flood Reports from/to the mobile application.

The system for detecting and forecasting natural disasters based on IoT [11] was modeled with the aid of the ns-3 simulator and compiled with the IoT standards available in the simulator. The authors in [12] describe the design and implementation of a real-time monitoring system for hydrologic applications. The proposed monitoring system presents useful characteristics such as large network capacity, sensor hardware compatibility, low power consumption, low cost, and minor impact on the natural environment.

There are many low-cost early warning systems like EWIN; one of them is “FLOOD MONITORING AND ALERTING SYSTEM” [13]. It shares some similarities, although key features of the systems are different, like the alert trigger method and the precision of the sensors used. It is observed that EWIN might offer a superior approach, since it uses faster communication protocols and a centralized information system. In another similar work, “The Implementation of an IoT-Based Flood Alert System” [14], the alert processing is made at each node. The node processing power can be a handicap in comparison with the proposed system that uses a dedicated server for such task. In the state of Tabasco, Mexico, attempts have been made to develop early warning systems such as the “Water Level Meter for Alerting Population about Floods” [15]; in this case, only laboratory experimental tests are presented. EWIN is one step ahead—it has been tested in real hydrometeorological phenomena and the system has worked in a favorable way, as shown in this document. A detailed comparison of various early warning systems is found in the paper “Computer Vision and IoT-Based Sensors in Flood Monitoring and Mapping: A Systematic Review” [16]. EWIN follows the latest methods and recommendations for this type of system; it can be placed on par with the latest flood alert systems that use IoT technology.

EWIN is an early warning system, adapted for the City of Colima, developed to alert the population of a possible overflow of the rivers, through the reading of hydrometeorological variables.

The elements that help early warning are:

RiverCore: They are fixed nodes and their main function is to measure the depth of the river in real-time.

Drifters: They are mobile nodes; these are released into the river when there is a possible overflow; their contribution to the system is to better understand the behavior of the river in these events.

Weather stations: These measures, in real-time, the variables: solar radiation, precipitation, vapor pressure, relative humidity, air temperature, humidity sensor temperature, barometric pressure, horizontal wind speed, wind gust humidity, wind direction, tilt, lightning strike count, and lightning average distance. These will be used in the future to complement the prediction model and allow more time for a possible evacuation.

Sniffer: System mountable to any vehicle—it can be a drone—this is used in the recovery process of drifters after a hydrometeorological event and obtain the data collected from it.

Server: Allows data archiving, data processing, and runs the web user interface of the platform.

The EWIN platform uses:

(1) Internet of Things (IoT) technology in each of the nodes.
(2) LORA (LOng RAnge) technology to communicate between drifters, RiverCore nodes, and the hardware located in the sniffer.
(3) MQTT (MQ Telemetry Transport) protocol to carry out the communication between the fixed and mobile nodes with the server in the cloud, making efficient handling of data acquisition.
(4) JSON (JavaScript Object Notation) protocol for data exchange.
(5) Low Energy Consumption Telemetry.

## 2. Materials and Methods

The components of the system include a fixed node (RiverCore), a mobile node (Drifter), a drone mountable sniffer, and weather stations, along with a web-based data acquisition platform, all integrated with IoT techniques to retrieve data through 3G cellular networks.

**Figure 1:**
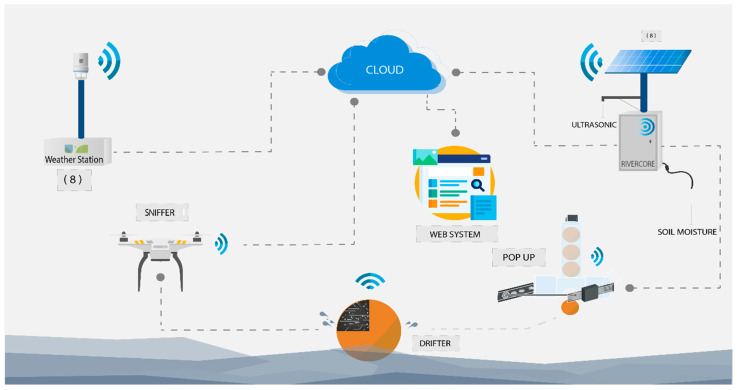
Elements of the early warning system (RiverCore, weather station, drifter, and sniffer).

The developed architecture uses the Message Queuing Telemetry Transport Protocol (“MQTT is an OASIS standard messaging protocol for the Internet of Things (IoT)” [17]), to send real-time data packages from fixed nodes to a server that stores retrieved data in a non-relational database. From this, data can be accessed and displayed through different customizable queries and graphical representations, allowing future use in flood analysis and prediction systems. The parts of the system can be seen in Figure 1.

### 2.1. System Components

RiverCore—a static low-cost gauging station for areas with cellular radio coverage.

**Figure 2:**
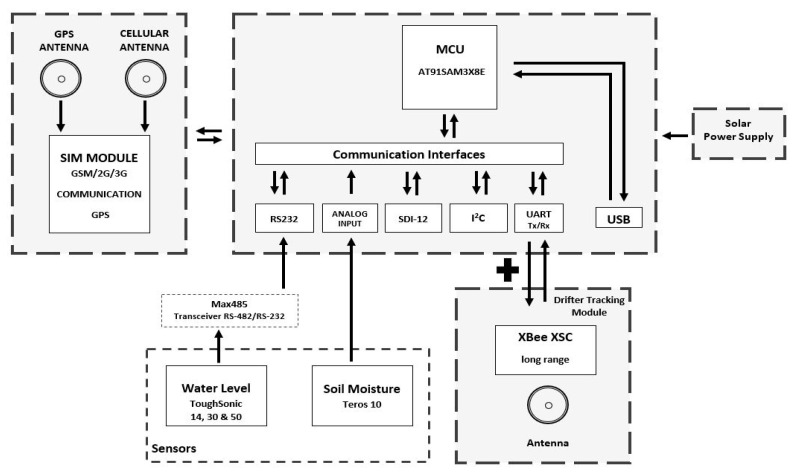
RiverCore hardware block diagram.

RiverCore fixed node hardware is composed of several components that allow it to retrieve data from sensors and send it to the data acquisition platform through the cellular network. It uses a 32-bit AT915AM3CU microcontroller, based on open-source hardware design. Months of testing prove its capabilities and perfect fit in the fixed node. Subsequently, the ultrasonic sensor originally used was replaced by a Toughsonic Water Level Sensor, and a soil moisture sensor was added to achieve more robust and precise readings. The fixed nodes communicate with mobile nodes (drifter) through XBee or LoRa radio interchangeably (“LoRa is a proprietary spread spectrum modulation scheme that is derivative of Chirp Spread Spectrum modulation (CSS) and which trades data rate for sensitivity within a fixed channel bandwidth.” [18]). To accommodate the new hardware, a daughterboard was designed to serve as a physical connection interface between the microcontroller board, sensors, RS-485 transceiver, and wireless radios. The hardware architecture can be seen in Figure 2.

The RiverCore fixed node is physically composed of a 32-bit microcontroller unit, a 3G cellular modem electronic board, an XBee (802.15.4) (“modules seamlessly interface with compatible gateways, device adapters and range extenders, providing developers with true beyond-the-horizon connectivity.” [19]), or LoRa radio, shield/daughterboard, an RS-485 transceiver, a regulated power supply, a solar charge controller, and a 12 v 80 A h battery.

**Radio link budgets**
  XBEE XSC PRO
    Transmit power 20 dBm.
    Receiver sensitivity −106 dBm.
    Spread spectrum FHSS.
    Outdoor/line-of-sight range 6 mi.
    Urban range 1200 ft.
  SIM5320A
    Receive sensitivity −106 dBm.
    Transmission power 33 dBm.

The weather station will be an important part of the forecasting model in the future, adding the variables it measures to be able to predict floods with more time.

It is intended to study the variables and compare them with the historical data of past hurricanes that have caused the overflow of rivers in the city. They are presented as part of the early warning system solution so that it is fully known.

**Figure 3:**
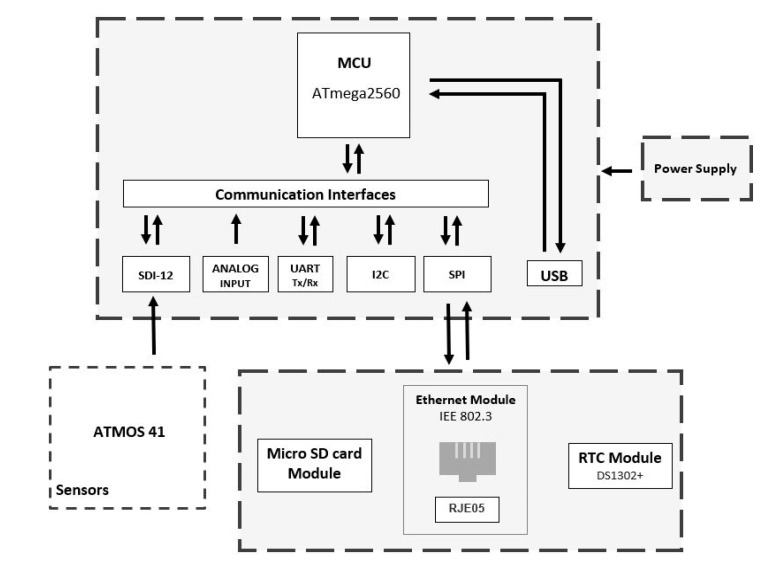
Weather station hardware block diagram.

This node (diagram shown in Figure 3) integrates a series of electronic components that the ATMOS 41 (all-in-one weather station [20]) weather station employs. Its high-performance, low-power Microchip 8-bit AVR RISC-based microcontroller handles the data of the 12 weather sensors included on ATMOS 41. The data are packed and sent to the platform via a wired internet connection. They also get stored with a timestamp on a Micro SD card as a backup.

**Weather Station Specifications**

Solar radiation: Range: 0 to 1750 W/m^2^; resolution: 1 W/m^2^; accuracy: ±5% of typical measurement.

Precipitation: Range: 0 to 400 mm/h; resolution: 0.017 mm; accuracy: ±5% of measurement from 0 to 50 mm/h.

Vapor Pressure: Range: 0 to 47 kPa; resolution: 0.01 kPa; accuracy: varies with temperature and humidity, ±0.2 kPa typically below 40 °C.

Relative Humidity: Range: 0 to 100% RH (0.00–1.00); resolution: 0.1% RH; accuracy: varies with temperature and humidity, ±3% typical RH.

Air temperature: Range: −50 to 60 °C; resolution: 0.1 °C; accuracy: ±0.6 °C.

Humidity sensor temperature: Range: −40 to 50 °C; resolution: 0.1 °C; accuracy: ±1.0 °C.

Barometric pressure: Range: 50 to 110 kPa; resolution: 0.01 kPa; accuracy: ±0.1 kPa from −10 to 50 °C, ±0.5 kPa from −40 to 60 °C.

Horizontal wind speed: Range: 0 to 30 m/s; resolution: 0.01 m/s; accuracy: greater than 0.3 m/s or 3% of measurement.

Wind gust: Range: 0 to 30 m/s; resolution: 0.01 m/s; accuracy: greater than 0.3 m/s or 3% of measurement.

Wind direction: Range: 0° to 359°; resolution: 1°; Accuracy: ±5°

Tilt: Range: −90° to +90°; resolution: 0.1°; accuracy: ±1°.

Lightning strike count: Range: 0 to 65,535 strikes; resolution: 1 strike; accuracy: variable with distance, >25% detection at < 10 km typical.

Lightning average distance: Range: 0 to 40 km; resolution: 3 km; accuracy: variable [20].

Drifter—A free-floating sensor that takes local measurements and tracks the flow in water systems. Drifters compile water measurements to estimate the flow of a hydrodynamic system; however, it requires communication capabilities and a method to integrate the gathered data into an appropriate data structure.

**Figure 4:**
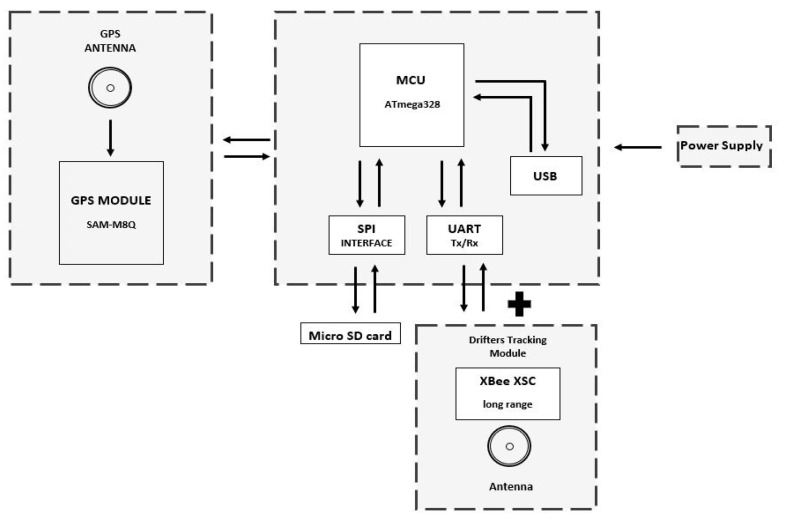
Drifter hardware block diagram.

The drifter architecture (Figure 4) includes a radio to communicate location information to the fixed node and RiverDrone (sniffer), thereby increasing the possibilities of finding and recovering them. It also incorporates an SD card module to store the location, speed, and potentially, any other data that are registered. Importantly, it can be equipped with additional modules or sensors. The system recovery method is explained in Section 3.2.

**Figure 5:**
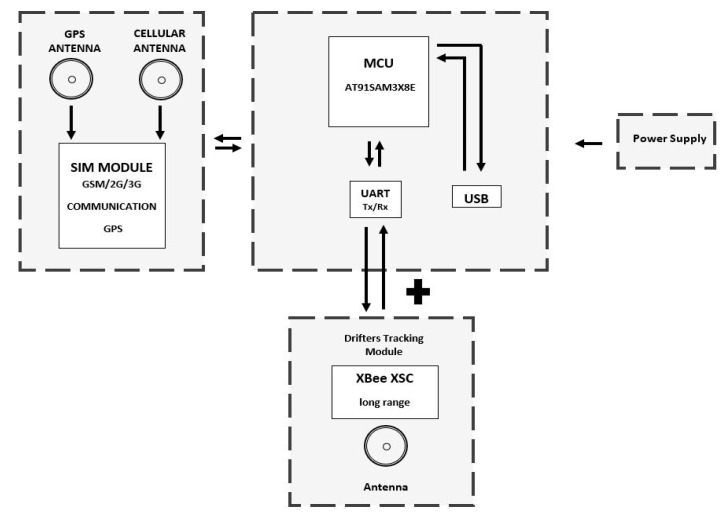
Sniffer hardware block diagram.

The sniffer architecture (Figure 5) allows the device to work as a sniffer looking for drifters. Equipped with matching radio, cellular communication, and GPS, drifters can report their location to the EWIN platform, where the information is forwarded and displayed on a web page.

### 2.2. Hardware Development

Hardware requirements have been defined according to the specific needs of the application, which has resulted in the development of fixed, weather station nodes, and drifter nodes, along with a data acquisition process that uses IoT techniques to retrieve data through the 3G cellular network.

**Figure 6:**
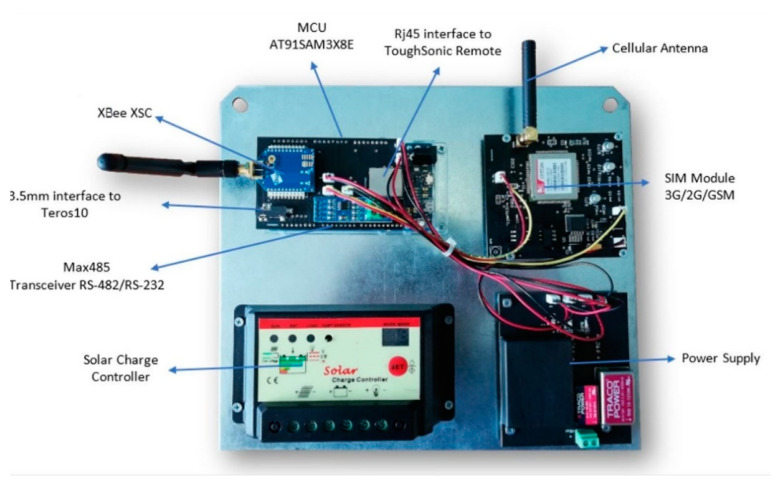
RiverCore hardware modules.

Power management, the cellular communication method, and other components described in a previous paper [21] remain unchanged. Their initial design effectively handles the extra load and demands of the new hardware now incorporated on the fixed node. The main hardware modules or the fixed node can be seen in Figure 6.

### 2.3. Sensor and Processing Considerations

The ultrasonic water level sensors ToughSonic Remote 14, 30, and 50 are used to measure the distance between the water surface and the sensor location. The data generated are then processed within the microcontroller and encapsulated in a JSON (“JavaScript Object Notation is a lightweight data exchange format”) structure, along with an identification string. These data are then transmitted to the server through the 3G cellular network and stored into the NoSQL (“No Structured Query Language”) database to carry out future calculations that can later be used for flood forecasting.

Several versions of this sensor are available, each of which possess different ranges and communication interfaces. Three different models were considered (ToughSonic Remote 14, 30, and 50), each with a different range, although all of them communicate through the RS-485 standard. The RiverCore processor board does not support this standard. Consequently, a Max 485 transceiver was employed to handle the communication between the microcontroller and the sensor. Before connecting to RiverCore, each sensor needs to be configured employing the manufacturer’s software, ASCII (“American Standard Code for Information Interchange”) streaming, with a 57,600 baud rate, which is the default configuration the fixed node accepts for this sensor. Some calculations to determine the distance between the ToughSonic sensor and the water occur in RiverCore (fixed node). The water depth is then calculated at the EWIN server with the characteristics of each site. Each sensor and site are treated differently since the formula varies between Remote 14, 30, and 50, and every physical site has its characteristics and topographic data.

Along with the ultrasonic water level sensor, every fixed node also incorporates a soil moisture sensor. The TEROS 10 is an analogue sensor, which makes its integration into the network simpler. However, an analogue sensor may also report some inconsistent values because analogue signals suffer from greater interference and other physical factors like the wiring length.

The TEROS 10 reports an output of 1000 to 2500 mv, which the microcontroller translates into voltage accordingly to its 12-bit resolution. These data are then transmitted, as is, and the server transforms the data into usable information regarding soil conditions.

The information from the humidity sensor is used to calibrate the hydrological model, since the soil has an antecedent humidity before a heavy precipitation episode. If the soil moisture is low, it has a greater capacity to absorb water volume and the opposite is true if the soil has a high moisture content. In this last scenario, it is easier to generate runoff. Therefore, the humidity sensor data are used to calibrate the hydrological model to know the response of the basin and to use these variables in the forecast model.

There are two types of fixed nodes on the EWIN network: RiverCore and weather stations. The weather station node retrieves the weather information from the ATMOS 41 weather station which packages 12 weather sensors. It features a 3-wire interface following the SDI-12 protocol for communicating sensor measurements.

The weather station measures 12 weather variables including air temperature, relative humidity, vapor pressure, barometric pressure, wind speed, wind gusts and direction, solar radiation, precipitation, lightning strikes, and lightning distance. The data are backed up internally by the weather station node and sent to the server unmodified to be stored and displayed.

The RiverCore main processing board is based on the AT91SAM3x8E, which can communicate with different protocols; this allows it to be compatible with different sensors and devices, with optimum efficiency.

The compatible communication protocols are listed below:

- I2C;
- SDI-12;
- SPI;
- RS-232/RS-485;
- USB;
- Analogue input.

The actual implementation of the RiverCore fixed node, which has been deployed in the experimental network, has inbuilt libraries for RS-232, Analog, I2C, and SDI-12 devices; however, different libraries can be developed to include compatibility with a wide range of hydrologic devices.

**Figure 7:**
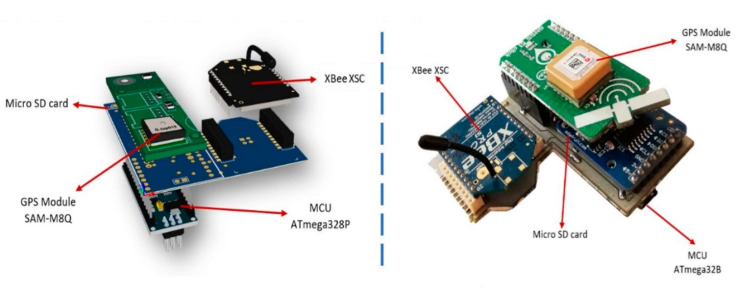
3D model and picture of drifter electronic components.

The drifter node integrates a GPS module, which retrieves location, time, and speed variables. It also contains a physical storage card slot to retrieve measured data. This device is sealed inside a waterproof enclosure that holds a magnetic switch, which powers the data logging mechanism while it flows through the river basins. These data are stored inside the MicroSD card and can be analyzed by the EWIN data acquisition platform. Drifter nodes add radio communication capabilities to transmit information to other devices like a fixed node and a sniffer. The above-depicted hardware components are shown in Figure 7.

The electronic components are sealed into a spherical lightweight plastic container using polypropylene material. The device’s mainboard is 8 cm long and 5.1 cm high; a bright color is recommended to facilitate the search and recovery of the equipment.

The drifting device operates using a magnetic, normally closed switch, allowing it to be turned on after it is sealed by removing a previously attached magnet. Current river conditions present challenges due to constant changes in current velocities, water level, the presence of vegetation, rain, and other environmental factors. Drifters have been used in many countries, making them a popular platform used to collect river parameters, such as the direction of the river’s water flow path. The proposed technological solution uses a drift network through various sections of the river. Similar technology solutions were published in [22,23,24,25,26,27].

The drifter has the possibility of integrating other sensors to measure variables such as water quality or depth.

The RiverDrone platform is a combination of software and hardware aimed to be mounted onboard other mobile exploring methods, such as drones (UAV) or other mobile infrastructure, adding an aided method to find as many drifters as possible when deployed. The RiverDrone hardware can be mounted on any drone that can support a weight of 400 g. The type of drone is not described because RiverDrone hardware is not connecting to the drone hardware at this time. The software constantly broadcasts a message searching for drifters. When a drifter is found, the software will report the sniffer and drifter location to the EWIN web platform via the 3G cellular network using MQTT communication protocol.

Natural water channels like rivers have many obstacles, cavities, and uneven shapes that may cause the drifters to get stuck; therefore, a tool like the RiverDrone is required to facilitate the retrieval process of such devices.

**Figure 8:**
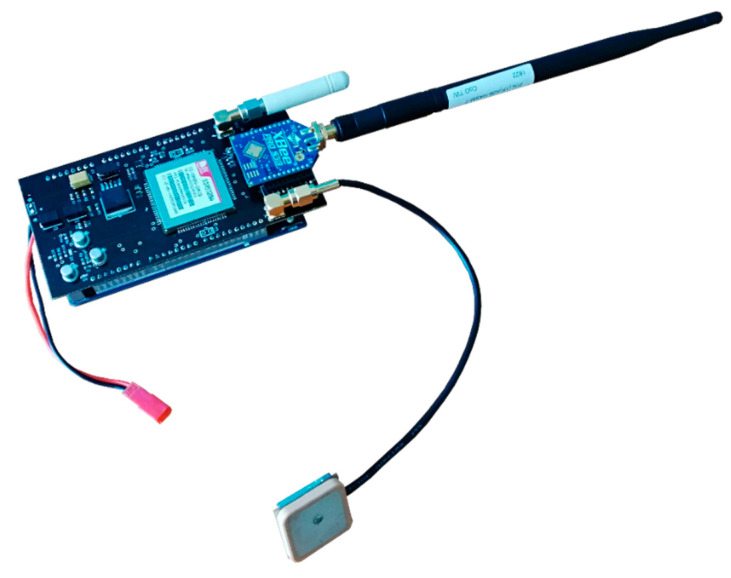
Sniffer hardware components.

The sniffer is physically composed of five different devices: a microcontroller unit, a 3G cellular modem electronic board, an XBee (802.15.4) or LoRa radio, a regulated power supply, and a LiPo battery (Figure 8). It contains the proper materials and components to localize the mobile nodes throughout the river and send these data to the server (Drifter Id, Latitude, Longitude, RSSI (“Received Signal Strength Indicator”), and Speed).

### 2.4. Alert System

The flow of a river undergoes variations over time, being subject to changes derived from its natural dynamics, as well as by human actions. For this reason, the topographic surveys that were carried out are necessary to obtain the flow using the Manning equation [28], and continuous monitoring of the water level to generate an accurate and reliable alert system. The design of the early warning level for a channel overflow is obtained by estimating the quantiles based on the maximum flow.

The alert system is based on thresholds obtained from several hydraulic simulations with different scenarios through terrain elevation models, since a lot of computing power and time is required to perform a simulation. A method based on the real-time water level is used and instead of simulating each measurement, thresholds were set as a semaphore—green when the water level is at less than the 50% of the capacity of the river, yellow if the water level is between 50% and 75% of the capacity, and red if it exceeds 75% of its capacity. This allows it to alert population in real-time.

Alert levels are calculated based on the flow measurement for each node. Every time the alert level changes state, it generates a notification in the system and sends an email notification to users with access to the system. Notifications change dynamically according to the risk of flooding. When the system estimates a higher risk, it will trigger the notifications with higher frequency, continuously alerting users to possible flood risk.

The time between the alert and potential flooding can vary according to the location of the sensor that generates the alert and the distance with the community alerted, however, a good estimated value is 10 min, enough for people to take shelter or get away from the high-risk zones.

Hydraulic models normally need 3 inputs—a field survey either in cross-sections or in a triangle irregular network, a rainfall–runoff analysis for different return periods, and a set of initial conditions such as roughness and normal depth.

Field surveys were made to model the terrain through a Triangular Irregular Network [29] based on coordinated points. This is one of the main inputs for hydraulic models. Historical precipitation data were analyzed as well to obtain different scenarios for various return periods. These two elements are the basis of the hydraulic model which determines flooding zones based on the runoff path through the terrain.

The sites evaluated for flooding were only the sites where the sensors were installed; these sites are well defined hydraulic sections that function as a checkpoint for the level measured and can be used to calibrate water level/discharge curves.

One-dimensional hydraulics models are used broadly because of short processing time; this allows quick visualization of results such as water elevation, flow, velocity, and overflow in certain stations, but these models have some disadvantages, mainly the lack of precision in flooding zones and the lack of results between the stations added to the model.

Bidimensional models, on the other hand, consider terrain information as an entity divided by small interconnected squares that its values depend on the results of the squares behind it. This method can predict the direction of the flow and can identify flooding zones based on the velocity and depth in more refined criteria; however, the modeling time is extended considerably, with a single run taking up to 20 h to complete.

**Figure 9:**
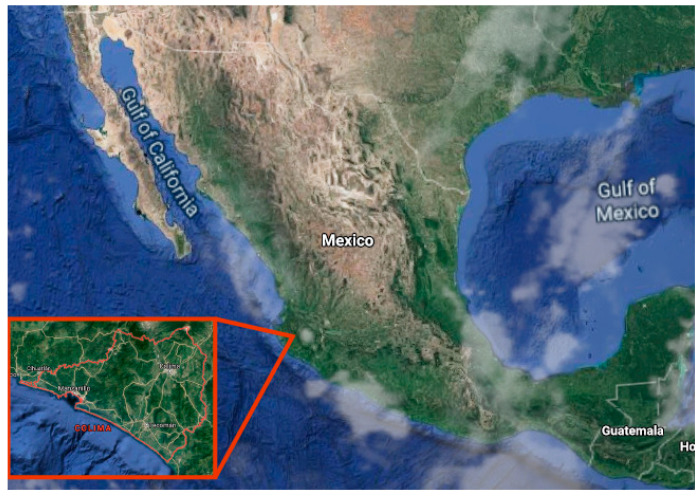
Zoom in of the state of Colima, in México.

Figure 9 shows the location of Colima México, where the study and data collection were carried out for this research.

**Figure 10:**
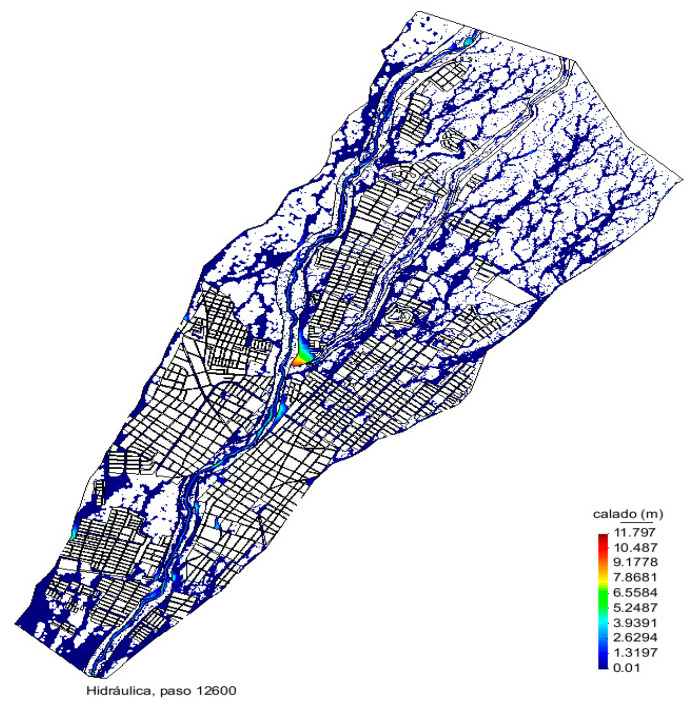
Rendered frame of the hydraulic model used to determine the overflow alert levels and zones.

The simulation of the model in Figure 10 presents the rivers Colima and Gertrudis monitored within the city of Colima, showing the existing depth in the rivers, to determine the flow of the water and the point of loss.

The color scale shows high depth in red color and low depth in blue color; the zones with high depth match are indeed problematic in real events, which shows the model is accurate.

The following section provides a discussion of the principle aspects of the software that maintain network functioning.

Simplified alerting system methodology.

(1) Simulation of the hydraulic model to obtain the overflow zones and levels around the area of interest.
(2) Topographic survey of each section of the river where a node will be placed.
(3) Determine the corresponding formula according to the characteristics of the terrain.
(4) Deploy RiverCore at the point of interest.
(5) Input characteristic of each section to the server.
(6) Process each data package sent by the installed RiverCores to determine the alert level.
(7) The information gets displayed on the web interface and an alert is triggered if it corresponds; in this case, the local authorities will be automatically notified.

### 2.5. Embedded System Software

To establish communication between hardware components and the cloud platform, which is installed on a dedicated server, it is necessary to build data packets. This is done by coding a certain set of instructions that permits the data to flow as is needed to send and receive adequate information.

**Figure 11:**
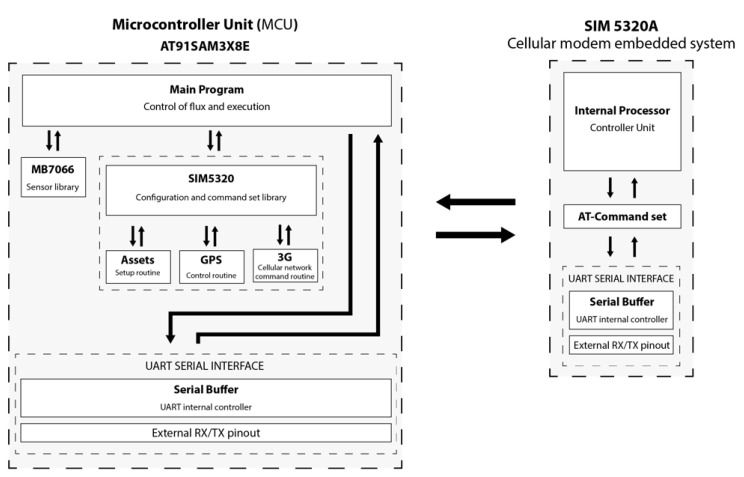
RiverCore firmware architecture.

As shown in Figure 11, the processing board is programmed with the main firmware, inside which different sets of libraries were developed for its correct functioning. The first set of libraries corresponds to the ultrasonic water level sensor which contains the code needed to carry out operations to measure the distance between the sensor and the riverbed. The second set of libraries contains the commands needed to establish a connection between the processing board and the cellular modem board. This set is divided into three libraries: the first one includes instructions for the setup routine of the modem, the second one belongs to GPS commands, and the third one includes the 3G connection commands that are needed to send packets through the cellular network.

At the beginning of the program, the processing board sets up the hardware environment to connect to the cellular network, then it retrieves GPS and water level sensor data, and finally, it forms a JSON string to send it through the 3G cellular network.

All the instructions used as AT commands are stored in cellular modems’ memory, as a command set. All the communication between processing and modem boards is made through the UART (Universal Asynchronous Receiver-Transmitter) serial protocol.

### 2.6. Drifter System Software

**Figure 12:**
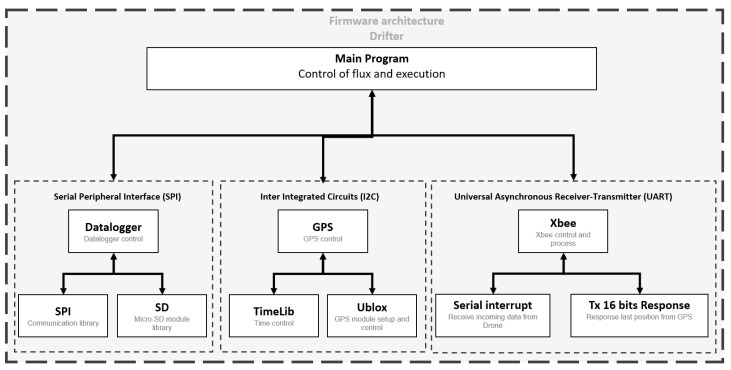
Drifter firmware architecture.

The objective of the implemented software architecture (Figure 12) is to gather GPS location information (altitude, latitude, longitude, and speed) to be stored in a micro SD card in compliance with a JSON format for further analysis.

Along with the data collection process, an interrupt routine monitors communications using XBee radio for data messages (frames) from RiverCore (RCFX). When a message is detected, the drifter responds with an RCFX frame. At the same time, sniffers also search for a message from the RiverDrone (RCDN) and the drifter responds with a frame containing: altitude, latitude, longitude, speed, RSSI.

As previously mentioned, the drifter is a sensor that measures real-time speeds and river water levels in different sections and connects to Rivercore nodes through wireless technology. The EWIN platform has information from all RiverCore nodes that measure the water level and supplements it with the information provided by all drifters to generate more accurate information.

### 2.7. Sniffer Embedded System Software

**Figure 13:**
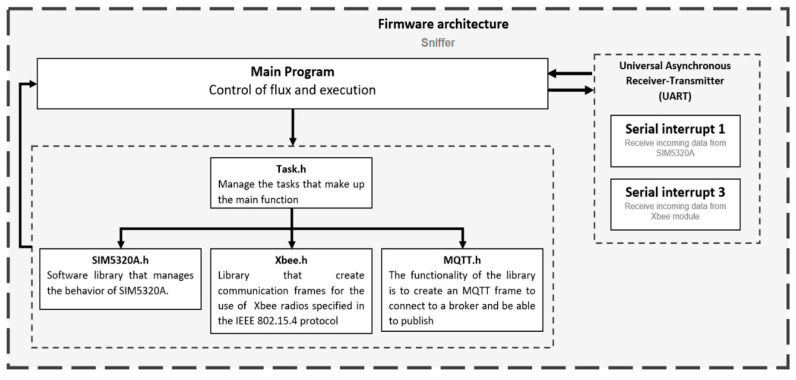
Sniffer firmware architecture.

The software running onboard is responsible for locating drifters by sending an 802.15.4 data frame in broadcast so that each drifter in range responds to the request. The response message of the drifter contains GPS location, speed, and signal strength of the XBee radio. The signal strength can be used to estimate the distance between the drifter and the sniffer (Figure 13).

The embedded software processes are found in Appendix A.

### 2.8. Responsive Web-Based Data Acquisition Platform

Along with EWIN devices, an IoT Cloud Platform has been developed to manage data acquisition from the RiverCore node environment. This cloud platform allows users to set-up and monitors the incoming data of the multiple deployed sensors.

The EWIN data acquisition environment is integrated by hardware devices, an MQTT Mosquitto Broker [30], and a NoSQL document-based data structure.

The EWIN dataloggers retrieve information from environmental variables and send it through a 2G/3G cellular network, using AT commands and the MQTT protocol, to a Linux server, which has an MQTT Mosquito Broker installed. This broker receives all the messages published to the topics, while a background Node.js script saves data using a MongoDB [31] document structure.

**Figure 14:**
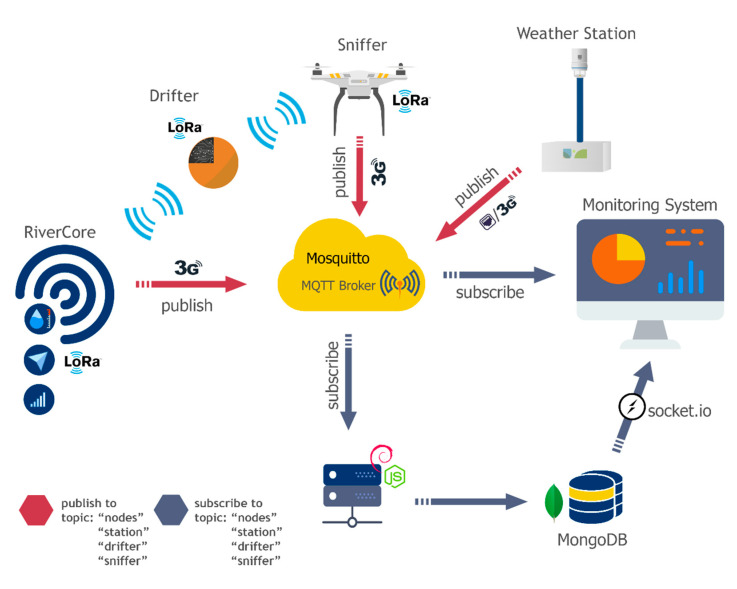
Data flow and technologies used on the data acquisition system.

Figure 14 describes the interaction between the main parts of the EWIN network now developed and deployed.

**Figure 15:**
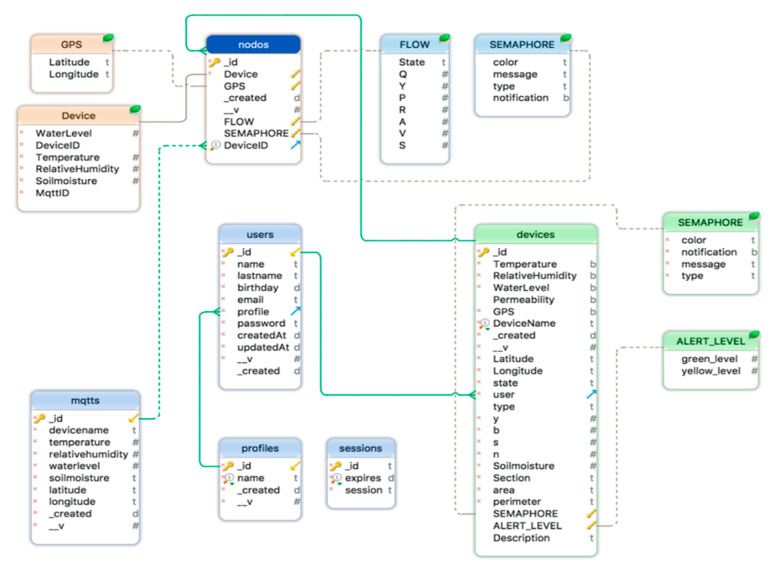
Database structure.

While information is saved in a MongoDB database, it can be retrieved using an online monitoring web platform, which listens to WebSocket (“is a computer communications protocol, providing full-duplex communication channels over a single TCP connection” [32]) that receives MQTT messages. Although the database was developed in a NoSQL environment, it was represented with a relational structure to make it easier to understand and interpret (Figure 15).

The backend server has been recently migrated to datacenter-grade hardware, and the platform has been mounted on a fresh install of the operating system. In addition to the main operating modules of the system, some other functions have been added, including the ability to back up the database to an offsite service like Amazon Web Services (“Amazon’s cloud services provider” [33]), Microsoft Azure (“Microsoft’s cloud services provider” [34]), or even a secondary physical server.

The web application consists of two central parts—the public part which shows the last 10 min of sensing and the private part, where persons can see the information in real-time and download the data stored on the server.

#### 2.8.1. Main Dashboard

The private web platform http://tairda.siteldi.mx/ integrates a dashboard to see retrieved data from all RiverCore nodes. The dashboard shows real-time data of the deployed nodes, a chart of current and previous data, and a map with markers which show the position of each node registered on the platform. Such markers change color according to the warning level of the corresponding river section. Fixed devices are registered and managed on a dedicated section of the web platform, where parameters such as the node name and sensor features are configured.

A similar section has been created to show the data captured by the weather station nodes. On both dashboards, clickable icons can be seen on a map representing the real-time position and status of the stations. Some variables are graphed in real-time, but, importantly, all data from every node are stored and can be retrieved in the historical section.

#### 2.8.2. Historical Data Section

The platform also allows users to recover historical data as a report, which shows data tables of all nodes filtered by name, date, device, etc. This information can then be displayed and downloaded as a PDF, CSV, or Excel file for further processing and analysis.

#### 2.8.3. Drifter Data Retrieving Section

The drifter section allows users to upload the JSON files generated by the drifters and displays the data in it, which consist of the distance travelled, number of samples, top speed, average speed, maximum altitude, and average altitude. By using the JSON format, different devices can publish useful weather data to the web system, regardless of the hardware used.

#### 2.8.4. Public Web Platform

**Figure 16:**
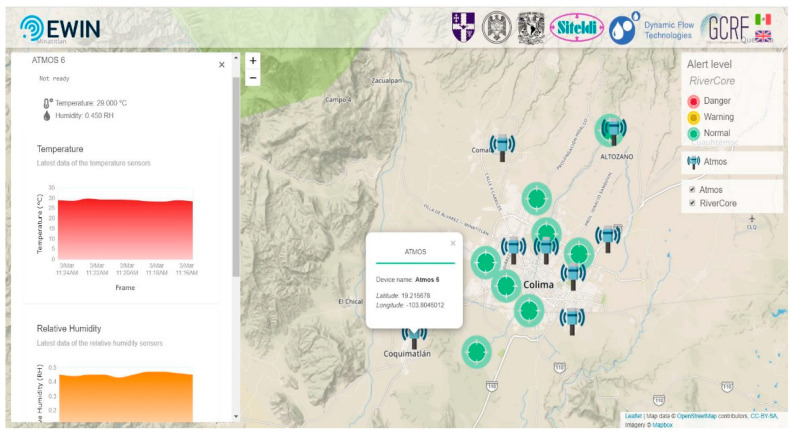
Public web platform.

The public version of the web platform http://tairda.siteldi.mx/ewin requires no authentication and only has one section (shown in Figure 16). A map is displayed with clickable icons representing every station and its status. When an icon is clicked, a sidebar displays a simplified version of the last few data packets received from the selected node. The site is intended to be shared and consulted by the general public.

#### 2.8.5. API REST

The interface permits live monitoring, historical data queries, and the administration of the different Emergency Water Information Network devices. The REST (“REpresentational State Transfer. It is an architectural style for distributed hypermedia systems” [35]) carries out the following functions:

(1) The RiverCore real-time monitoring section displays the variables received from the deployed sensors along with the flow calculated from these data, and the model obtained from the previous topographic work. The interface displays the information on charts and graphs for easy interpretation. It also contains a map where every network device is shown. The icons representing the RiverCore nodes on the map individually change colors (green, yellow, or red), according to the current state of the river. The overflow alert is based on the flow analysis developed by the water team of the project. When an alert is triggered, a notification is displayed in the web browser and added to the notifications list in the navigation bar of the platform.
(2) Device section: This section of RiverCore manages the fixed nodes and permits data queries. Only persons with administrator privileges can add, edit, and delete. When registering a fixed node, topographic information (allowing the platform to calculate flow and show alerts) and location must be included. The topographic information is unique to every location and the formula needed to display the correct information on the platform is also site-specific.
(3) Report section: This section displays a personalized query of records, generates a report, and downloads it in several user-selectable formats (Excel, PDF, and CSV).
(4) Drifter section: This section displays the real-time data related to the drifters. The data may come from the RiverCore or RiverDrone sniffers, which then are represented in tables and maps. It also allows users to upload the data retrieved from the drifter’s SD card.

#### 2.8.6. Independent Web Services

- RiverCore: Responsible for receiving the frames generated by RiverCore’s fixed devices, storing them in the database, and sending a message with the processed data to the socket server that is in the REST API.
- Drifters: In charge of receiving the frames of the drifter devices, storing them in the database, and sending a message with the processed data to the socket server that is in the REST API.
- Sniffer: Receives the frames of the sniffer devices, stores them in the database, and sends a message with the processed data to the socket server that is in the REST API.

## 3. Results

### 3.1. Network Results

#### Data Processing

**Figure 17:**
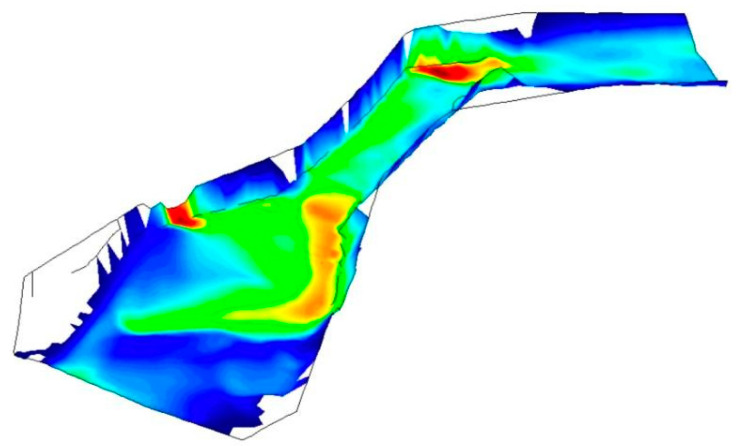
Example of a simulation which shows overflow in section.

The prediction process begins with the data of the sensors received on the server. Previously, a topographic model of each monitored section has been generated along with its simulation. This allows the generation of specific parameters to feed the system which will use them, and the corresponding formula, to generate overflow alerts. Figure 17 shows a simulation of a river section; the red color of the heat map indicates the overflow zones with simulated flow and conditions.

**Figure 18:**
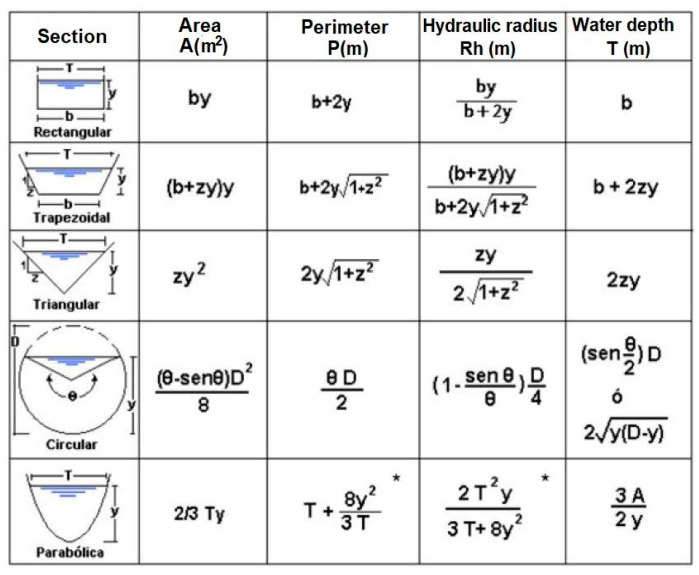
Formulas used to calculate flow accordingly to the canal shape.

The flow of the river can be calculated with well-known formulas that correspond to a certain shape of the canal. The correct formula is selected accordingly to the results of the simulated data of the topographic survey. This allows the server to calculate the flow in real-time and compare it with the previously determined levels; those were established in each node accordingly to the simulation and to the topographic measurements made based on each section. By this, the stream velocity and height could be properly determined taking into consideration the terrain’s geometry; therefore, a semaphore risk indicator was stablished using different alerts: (green—no risk; yellow—warning; and red—danger); that way, the system will be able to predict overflow in the river and possible flooding. Figure 18 shows a list of formulas used to obtain the flow based on the shape of the section.

During 2019, the EWIN network registered several important weather events. A couple of them are described below.

The EWIN web platform notifies its users when RiverCore data exceed pre-set water flow limits. On 10 September, an alert was triggered at node 14. This node, located just north of the city, is the northernmost node and is the closest to the Colima Volcano, where most floodwaters are generated. At the time, there was no rain downstream in the city. This event possessed the conditions necessary to cause a flash flood.

**Figure 19:**
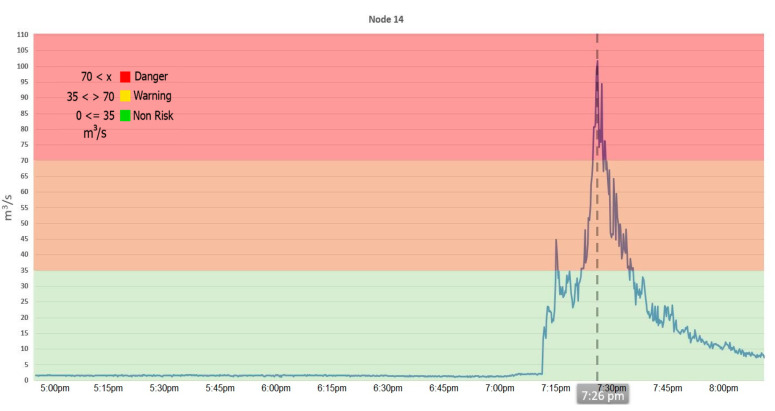
Calculated flow from data and its relationship with the precalculated alert level at node 14 on 10 September 2019.

Fortunately, no flash flood occurred, since the precipitation at the volcano, although intense, was of short duration, as shown in Figure 19.

The most remarkable weather event in the state of Colima during the 2019 rainy season was Hurricane Narda.

According to data from CONAGUA, on 19 September 2019, Narda generated heavy rains in several regions of Colima, Guerrero, Jalisco, Michoacán, and Nayarit; wind with gusts exceeding 70 km/h in Colima, Jalisco, and Michoacán; with gusts exceeding 60 km/h in areas of Guerrero and Nayarit, and high waves from 2 to 4 m on its coasts [36]. The reports of maximum punctual rain recorded in 24 h by this institution are 29 September: 342.3 mm and 30 September: 28.5 mm [37].

The Narda study was done as follows: first, the topographic survey of each area was carried out and then, the fixed nodes were installed; at that time, there were only four. One of them is the most important because it is the one found upstream. The water level data of each of the four operational nodes were collected on the server; before this, the information generated from the topographic surveys were fed to the hydraulic model and based on these alert levels were determined at each studied point. The system processed the data with the aforementioned formulas and compared them with the alert levels; at this point, an alert may be triggered accordingly. This process happens as soon as the data from any node arrive; usually every 60 s, each node reports new data to the server.

**Figure 20:**
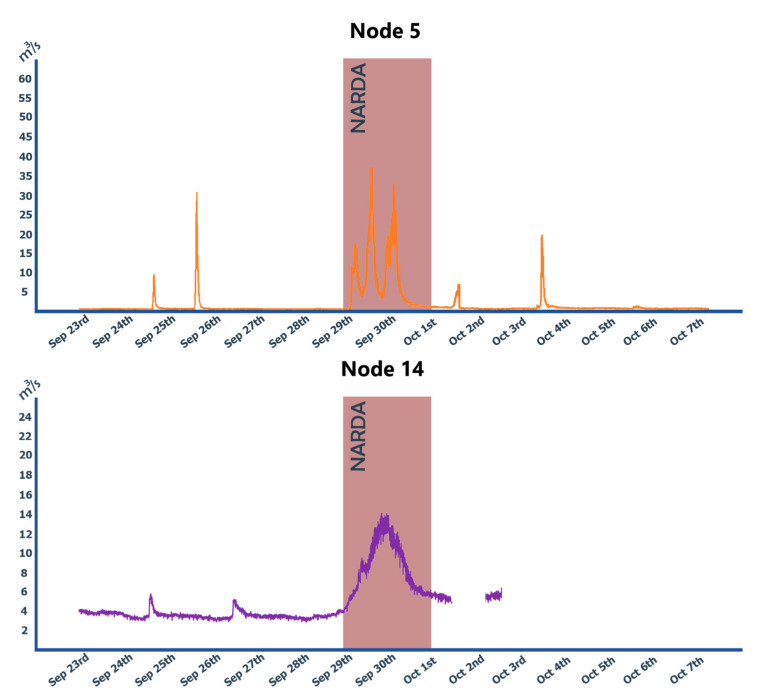
Calculated flow from data of nodes 5 and 14 during Narda hurricane, 23 September to 7 October 2019.

It is important to mention that no overflow risk was registered during the entire duration of the event. Figure 20 shows the behavior of the river level at some of the monitoring points. Fifteen days of data were graphed, including the mentioned storm. To obtain the information as it is presented, first, the existing data from the two nodes were extracted of the database, then a filtering was made to eliminate noise that can lead to false positives or false negatives. Around 4 to 5 percent of the original data had to be discarded due to inconsistencies, allowing the alerts to be raised only in the likelihood of an overflow of the monitored area.

### 3.2. Drifter Retrieval

**Figure 21:**
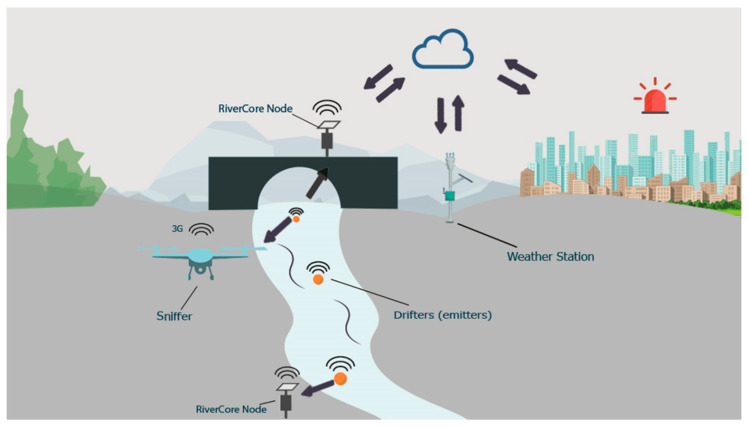
Drifter tracking system.

The proposed tracking solution is comprised of three types of devices shown in Figure 21: RiverCore fixed nodes, drifters, and a RiverDrone. This would work by detecting drifters while they pass through certain fixed stations that act as checkpoints. Then, as their location gets recorded by the data acquisition platform, a sniffer possibly mounted on a drone can be sent to search for them near the last activated checkpoint.

Drifters emit an 802.15.4 radio signal as they pass along the river path. When drifters pass below a RiverCore node, the signal is detected by a radio coordinator module which is attached to the fixed node so that the drifter location can be estimated in real-time.

When drifters get lost, it is possible to track their last location between shorter segments of the river. In this way, when it becomes necessary to search for drifters, can be released to follow their track, starting immediately after they were detected by a fixed node. The sniffer on board will scan for drifters using a radio coordinator module, so the location of drifters can be reported in real-time, using a cellular communication module.

The tracking system began field testing during the last trimester of 2019 and, currently, a second version using LoRa is under development. Both radio solutions will be tested to determine the best solution that suits the needs of the project.

When the sniffer detects a drifter, it sends the drifter’s GPS information to the server through 3G technology. If the drifter is detected by a fixed node, only its ID is sent to the server to confirm what has occurred in its trajectory up to that point.

## 4. Discussion

Several important aspects that can be improved were identified during the final stages of project evaluation. First, close consultation with hydrologists and data scientists will be needed to implement machine learning algorithms and other forecasting methods that can be used in a more extended network formed by many of these devices, thus permitting scalability. It is also worth mentioning that if this model is replicated, artificial canals should be considered to deploy the water depth sensors, hence they will not change their characteristics and the model will remain valid; otherwise, frequent topographic surveys of each sensing site will be necessary.

General maintenance is a must for this type of deployed hardware. Exposure to the environment can severely affect the functionality of the devices, and with that, the accuracy of the platform. The most affectable part of the fixed nodes are the ultrasonic sensors; any strange object in the sensing field will modify the data.

Today, cellular networks are well developed in most countries, providing a suitable platform upon which to deploy monitoring networks. The communications technology should be chosen according to the needs of each implementation. Due to the difficult terrain where the infrastructure was deployed, other communication technologies were analyzed and discarded; taking advantage of the infrastructure of the service providers in the area was the most practical solution. This does not minimize the fact that cellular networks can fail during natural disaster situations, as with any other wireless technology. Because most bodies of water bodies are not equipped with any sensing technology, despite the potential risks they can represent, this work represents an important step in using state-of-the-art technologies in the area of hazard prediction in conjunction with fluid dynamics research. A significant limiting factor in employing technological solutions is their cost. This work shows that a low cost and reliable water level monitoring network is, indeed, feasible. The average cost for the whole system is approximately 60,000 USD.

## 5. Conclusions

This document has presented an early warning system to alert areas that can suffer from flash floods in real time. Flash floods represent a serious natural hazard that a significant number of persons worldwide must face in terms of loss of life and long-term societal effects. This proposal offers a solution that integrates an early warning system, notifications, and real-time monitoring of flood risk areas. The platform was successfully implemented in the State of Colima, Mexico, covering the metropolitan area of Colima and Villa de Alvarez. This platform is comprised of eight hydrological monitoring stations, eight meteorological stations, mobile monitoring stations called “drifters”, and a sniffer to help in the location of the drifters.

The results show that this system is affordable, technologically functional, and provides the few minutes of warning. Having these types of systems in place, together with hydrological forecasts, can help communities, decision-makers, and government officials properly plan or avoid urban development in at-risk areas and provide persons with a few valuable minutes to evacuate and possibly save their lives.

Thanks to the data generated in this project, along with the coordination with the local authorities, we can conclude that a complete Emergency Water Information Network EWIN network may shortly reduce the impact of floods in the City of Colima, providing information for city planning and, in a worst-case scenario, offering citizens several minutes to evacuate from flash flood zones.

The presented system is still under development. The integration of the weather data into the warning model is a key feature yet to be implemented. In the future, the proposed method with the deployed hardware will continue improving adding precision and features suggested by the current users.

## Appendix A

### Appendix A.1. Embedded RiverCore Software Process

**1.—Setup:**
  1.1.—Configure microcontroller’s USB serial port baud rate.
  1.2.—Configure USART 1 baud rate matching the SIM5320A connection.
  1.3.—Configure USART 2 baud rate matching the ToughSonic Remote sensor connection.
  1.4.—Configure USART 2 baud rate matching the XBee or LoRa radio connection.
  1.5.—Configure Teros10 Sensor (analogue).
  1.6.—Configure SIM5320A initialization routine and PIN.

**2.—Tasks:**
  2.1.—Initiate routing (30 s).
  2.2.—Start the SIM5320A configuration routine, if it has already been started but not configured (70 s).
  2.3.—Begin the raw data gathering routine from the Teros10 sensor (30 s).
  2.4.—Search for drifters (sniffer) routine, it sends a broadcast message on a specific channel and PAN ID. A packet or frame transmit request 16-bit address (RCFX) is sent.
  2.5.—Publish information of both sensors through the MQTT telemetry protocol to the server.
  2.6.—Publish if a specific drifter has been detected.
  2.7.—Restart the routine if a call or an error message has been detected.

**3.—Serial interruptions:**
  3.1.—Serial 1—SIM5320A contains the configuration routine (start, setup, restart).
  3.2.—Serial 2—Obtains distance from the water level sensor.
  3.3.—Serial 3—Collects XBee 802.15.4 information. It stores and obtains information and verifies the “RCFX” frame response (which means a drifter has responded).
  3.4.—Clear buffer.

### Appendix A.2. Embedded Drifter Software Process

**1.—Setup:**
  1.1—Configure microcontroller´s Baud rate, matching with the XBee radio.
  1.2—Configure GPS (I2C—protocol).
  1.3—Configure Micro SD card reader (SPI-protocol).

**2.—Task (If there is a GPS signal):**
  2.1.—Obtain a GPS location.
  2.2.—Update date and time of the microcontroller.
  2.3.—Store information (date, time, altitude, latitude, longitude and speed) in JSON format into the MicroSD.

**3.—Serial interruptions:**
  3.1.—Obtain data from XBee 802.15.4 to then store and verify that it is a frame whose origin is RiverCore or RiverDrone.
  3.2.—Respond with a frame transmit request 16-bit address (RCFX) if the frame comes from RiverCore.
  3.3.—Respond with a frame transmit request 16-bit address (RCDN: altitude, latitude, longitude, speed, and RSSI) if the frame comes from RiverDrone.
  3.4.—Clean reception buffer.
  3.5.—Clean transmission buffer.

### Appendix A.3. Embedded Sniffer Software Process

**1.—Setup:**
  1.1.—Configure microcontroller’s USB serial port baud rate.
  1.2.—Configure USART 1 baud rate matching the SIM5320A connection.
  1.3.—Configure USART 3 baud rate matching the XBee 802.15.4 radio connection.
  1.4.—Configure SIM5320A initialization routine and PIN.

**2.—Tasks:**
  2.1.—Initiate routine (30 s).
  2.2.—Start the SIM5320A configuration routine, if it has already been started but not configured (70 s).
  2.3.—End a broadcast on a specific channel and PAN ID. A packet or frame transmit request 16-bit address (RCDN, 20 s) is sent.
  2.4.—Obtain RiverDrone location by reading the internal GPS of the SIM5320A (15 s).
  2.5.—Publish information about the location of the sniffer (60 s).
  2.6.—Publish the requested information if a drifter has been detected. (60 s).
  2.7.—Restart routine if a call or an error message has been detected.

**3.—Serial interruptions:**
  3.1.—Serial 1—SIM5320A contains configuration routine (start, setup, restart).
  3.2.—Serial 3—Obtains XBee 802.15.4 information. Stores and obtains information, verifies the “RCDN” frame response (which means a drifter has responded), publishes it as mentioned in task 2.6.
  3.4.—Clear buffer.

## References

[1] Centre for Research on the Epidemiology of Disasters (2015). The Human Cost of Weather-Related Disasters; 1995–2005.

[2] Louvain C. (2019). Natural Disasters 2018.

[3] Lehner B., Döll P., Alcamo J., Henrichs T., Kaspar F. (2006). Estimating the impact of global challenge on flood and drought risks in Europe: A continental, integrated analysis. Clim. Chang..

[4] Li Y., Grimaldi S., Walker J.P., Pauwels V. (2016). Application of remote sensing data to constrain operational rainfall-driven flood forecasting: A review. Remote Sens..

[5] Alfieri L., Thielen J. (2015). A European precipitation index for extreme rain-storm and flash flood early warning. Meteorol. Appl..

[6] Thielen J., Bartholmes J., Ramos M.H., De Roo A. (2009). The European Flood. Alert System—Part 1: Concept and development. Hydrol. Earth Syst. Sci..

[7] Bartholmes J.C., Thielen J., Ramos M.H., Gentilini S. (2009). The European Flood Alert System EFAS—Part 2: Statistical skill assessment of probabilistic and deterministic operational forecasts. Hydrol Earth Syst. Sci..

[8] Smith A., Sampson C., Bates P. (2015). Regional flood frequency analysis at the global scale. Water Resour. Res..

[9] Hoedjes J.C.B., Kooiman A., Maathuis B.H.P., Said M.Y., Becht R., Limo A., Mumo M., Nduhiu-Mathenge J., Shaka A., Su B. (2014). A Conceptual Flash Flood Early Warning System for Africa, Based on Terrestrial Microwave Links and Flash Flood Guidance by Joost Geo-Inf. ISPRS Int. J..

[10] Burkard S., Fuchs-Kittowski F., Muller R., Pfutzner B. Flood Management Platform for Small Catchments with Crowd Sourcing. Proceedings of the IEEE 2018 5th International Conference on Information and Communication Technologies for Disaster Management (ICT-DM).

[11] Rossi C., Favenza A., Scullino F., Macchia V., Spoto G.L., Dominci F. Evaluating FLOODIS: Mobile sensing for a flood emergency service in the cloud. Proceedings of the IEEE: 2015 International Conference on Cloud Technologies and Applications (CloudTech).

[12] Marin-Perez R., García-Pintado J., Gómez A.S. (2012). A real-time measurement system for long-life flood monitoring and warning applications. Sensors.

[13] Priya S.J., Akshaya S., Aruna E., Julie J.A.M., Ranjani V. (2017). Flood Monitornig and Alerting System. Int. J. Comput. Eng. Technol..

[14] Shah W.M., Arif F., Shahrin A.A., Hassan A. (2018). The Implementation of an IoT-Based Flood Alert System. Int. J. Adv. Comput. Sci. Appl..

[15] Hernandez-Nolasco J.A., Wister Ovando M.A., Acosta F.D., Pancardo P. Water Level Meter for Alerting Population about Floods. Proceedings of the 2016 IEEE 30th International Conference on Advanced Information Networking and Applications (AINA).

[16] Arshad B., Ogie R., Barthelemy J., Pradhan B., Verstaevel N., Perez P. (2019). Computer Vision and IoT-Based Sensors in Flood Monitoring and Mapping: A Systematic Review. Sensors.

[17] Message Queuing Telemetry Transport (MQTT). http://mqtt.org.

[18] SEMITECH LoRa™ Modulation Basics. https://web.archive.org/web/20190718200516/https://www.semtech.com/uploads/documents/an1200.22.pdf.

[19] DIGI XBEE® 802.15.4 PROTOCOL COMPARISON. https://www.digi.com/pdf/xbee-802-15-4-protocol-comparison.

[20] Meter Group ATMOS 41. https://www.metergroup.com/environment/products/atmos-41-weather-station/.

[21] Moreno C., Aquino R., Ibarreche J., Pérez I., Castellanos E., Álvarez E., Rentería R., Anguiano L., Edwards A., Lepper P. (2019). RiverCore: IoT Device for River Water Level Monitoring over Cellular Communications. Sensors.

[22] Emery L., Smith R., McNeal D., Hughes B., Swick W., Macmahan J. Autonomous collection of river parameters using drifting buoys. Proceedings of the Oceans 2010 MTS/IEEE Seattle.

[23] Emery L., Smith R., McQuary R., Hughes B., Taylor D. Autonomous river drifting buoys—Applications and improvements. Proceedings of the OCEANS’11 MTS/IEEE KONA.

[24] Beard J., Weekly K., Oroza C., Tinka A., Bayen A.M. Mobile phone based drifting lagrangian flow sensors. Proceedings of the 2012 IEEE 3rd International Conference on Networked Embedded Systems for Every Application (NESEA).

[25] Postacchini M., Centurioni L.R., Braasch L., Brocchini M., Vicinanza D. (2016). Lagrangian Observations of Waves and Currents from the River Drifter. IEEE J. Ocean. Eng..

[26] Kadir H.A., Darus N.H., Admire I.K., Arshad M.R. Development of drifting buoy inspired by water strider for shallow water environment. Proceedings of the 2017 IEEE 7th International Conference on Underwater System Technology: Theory and Applications (USYS).

[27] Tinka A., Rafiee M., Bayen A.M. (2013). Floating Sensor Networks for River Studies. IEEE Syst. J..

[28] Mott R.L., Pedraza C.R.C. (1996). Mecánica de Fluidos Aplicada.

[29] Baghdadi N., Mallet C., Zribi M. (2020). QGIS y sus Aplicaciones en Agua y en Gestión del Riesgo.

[30] Eclipse Mosquitto ™ Open Source Message Broker. https://mosquitto.org/.

[31] MongoDB Document Database. www.mongodb.com.

[32] Wang V., Salim F., Moskovits P. (2013). The Definitive Guide to HTML5 WebSocket.

[33] Amazon Web Services. https://aws.amazon.com/es/what-is-aws/.

[34] Microsoft Azure. https://azure.microsoft.com/es-mx/overview/what-is-azure/.

[35] Patni S. (2017). Pro RESTful APIs: Design. Build and Integrate with REST, JSON, XML and JAX-RS.

[36] Conagua Aviso37-19. https://smn.conagua.gob.mx/files/pdfs/comunicados-de-prensa/Aviso37-19.pdf.

[37] Conagua Narda. https://smn.conagua.gob.mx/tools/DATA/Ciclones%20Tropicales/Ciclones/2019-Narda.pdf.

